# Targeted Hybrid Nanocarriers as a System Enhancing the Skin Structure

**DOI:** 10.3390/molecules26041063

**Published:** 2021-02-18

**Authors:** Agnieszka Lewińska, Marta Domżał-Kędzia, Kinga Kierul, Michał Bochynek, Dominika Pannert, Piotr Nowaczyk, Marcin Łukaszewicz

**Affiliations:** 1 Faculty of Chemistry, University of Wroclaw, Joliot-Curie 14, 50-383 Wroclaw, Poland; 2 Department of Biotransformation, Faculty of Biotechnology, University of Wroclaw, Joliot-Curie 14, 50-383 Wroclaw, Poland; marta.domzal@uwr.edu.pl (M.D.-K.); michal.bochynek@uwr.edu.pl (M.B.); 3 InventionBio Sp. z o.o., Wojska Polskiego 65 st., 85-825 Bydgoszcz, Poland; kinga.kierul@inventionbio.pl (K.K.); dominika.pannert@inventionbio.pl (D.P.); 4 Faculty of Health Science, University of Opole, ul. Katowicka 68, 45-060 Opole, Poland; piotr.nowaczyk@uni.opole.pl; 5 Dr. Nowaczyk Research and Innovation Center Sp. z o.o. Sp. K., ul. Żmigrodzka 81-83 lok. 205, 51-130 Wroclaw, Poland

**Keywords:** surfactin, levan, nanoemulsion, nanoparticles, skin, formulation, anti-aging

## Abstract

The skin is constantly exposed to external and internal factors that disturb its function. In this work, two nanosystems-levan nanoparticles and a surfactin-stabilized nanoemulsion were preserved (tested for microbial growth) and characterized (size, polydispersity, Zeta potential, and stability). The nanosystems were introduced in the model formulations-cream, tonic, and gel, and confirmed by TEM. The analysis showed that nanoemulsion has a spherical morphology and size 220–300 nm, while levan nanoparticles had irregular shapes independently of the use of matrix and with particle size (130–260 nm). Additionally, we examined the antiradical effect of levan nanoparticles and nanoemulsion in the prototype of formulations by scavenging DPPH (2,2-diphenyl-1-picrylhydrazyl; EPR spectroscopy). The model cream with both nanosystems and the whole range of products with nanosystems were evaluated in vivo for hydration, elasticity, smoothness, wrinkles and vascular lesions, discoloration, respectively. The cream improved skin condition in all tested parameters in at least 50% of volunteers. The use of more comprehensive care, additionally consisting of a tonic and gel, reduced the previously existing skin discoloration to 10.42 ± 0.58%. The presented prototype formulations are promising in improving skin conditions.

## 1. Introduction

The topical route of administration has many advantages for the treatment of various skin disorders as well as cosmeceutical purposes [1]. The demand for ever more effective treatments inspires scientists as well as industry to develop interdisciplinary solutions. The boundaries between pharmacy and cosmetics are blurring. Newly developed carriers of active substances are frequently examined in cosmetic formulations since it is easier and less bureaucratic than conducting detailed clinical trials. Consequently, obtained results can further be developed in pharmaceutical research. This concept is represented by the gradually increasing number of published articles in pharmaceutical journals. Standard cosmetic compounds are described as newly discovered for medicine in the treatment of various diseases, e.g., nanoparticles of hyaluronan are used in the treatment of skin inflammatory diseases [2], encapsulated curcumin tested for treatment of psoriasis and melanoma [3], coenzyme Q-10 encapsulated in microemulsion used to improve regeneration of skin [4]. The constant urge for better products forces development of sophisticated formulations, aiming to improve performance, appearance, and sensorial benefit and safety.

In the past few years, applied nanotechnology has received increasing attention in pharmaceutical, food, cosmetic, textile, personal care, agricultural, chemical, biotechnology, biomedical, and sensor industries. Colloidal carrier systems are advantageous in solubilizing poorly soluble active substances [5]. Biologically active substances’ encapsulation increases their stability and protects them from the harsh environment and thus saves them from degradation. The preparation and application of nanostructured systems have become an integral part of the development of cosmetology, providing significant advances in delivering active substances. Colloidal systems from various raw materials create polymer nanoparticles, micelles, solid lipid nanostructures, liposomes, nano-, and microemulsion, etc. [6,7]. By using distinct formulations and preparation methods, nanosystems can be incorporated into diverse types and formats of cosmetics.

Most cosmetics are applied topically. Therefore, an important aspect is the penetration ability through the skin barrier. The external layer stratum corneum is an obstacle to the penetration of active substances, especially hydrophobic. Due to encapsulation, hydrophobic compounds are easier to deliver to the deeper skin layers [8]. Encapsulation also has an economic dimension. Encapsulation enables significantly lower amounts of active substances, reducing the potential skin irritation effect, but still obtaining the desired effect. Therefore, cosmetic products containing nanocarrier can significantly improve their effectiveness, enabling overall lower concentration than “traditional” products, delivering a higher amount of active ingredients to the skin’s deeper layers.

One of the more desirable effects is skin hydration. For this purpose, nano-systems composed of moisturizing substances are used. One of the most commonly used moisturizing substances in cosmetics is hyaluronic acid (HA) [9]. However, conventional HA preparations are characterized by difficult penetration into the skin due to the large diameter of its particles. Another natural polymer is levan. It is not naturally present in the skin, but due to its properties, it can be a good candidate for HA replacement. The transepidermal water loss (TEWL) test, showed that the moisturizing effect of levan is comparable to that after using hyaluronic acid [10]. Levan is a fructose polymer synthesized by *Bacillus subtilis* and is one of the novel components which might improve skin condition. Levan provides deep and long-lasting skin hydration due to its porous structure. It is non-toxic to human fibroblasts and keratinocytes and has no hemolytic effect on erythrocytes. Our previous research has also shown that it has antioxidant properties [11]. Despite its high molecular weight, levan can be incorporated into a variety of active substance delivery systems [12]. Both of these compounds are polymers with large particle diameters, which makes it difficult for their penetration through the skin. Hence, their use in polymeric nano-systems can significantly affect their skin penetration possibilities. Polymeric nanoparticles (PNs) with their specific ability have attracted enormous attention. PNs are composed of amphipathic copolymers that tend to self-assemble into particles with unique architecture [13].

It is also very important to provide nutrients, the nanoemulsions are perfect as nanocarriers. Composed of a suitable surfactant, they ensure the formation of small vehicles with a bioactive substance inside [14]. The use of nanoemulsions is a very good solution, especially for hydro and lipophilic active substances. Their enclosure in a nanoemulsion protects them against the influence of the external environment and increases their stability, so they can be delivered deep into the skin while maintaining their activity. Surfactants are an essential component of the dispersion system and their presence improves the penetration of nanoemulsions through the stratum corneum. However, synthetic surfactants often cause allergies and irritate the skin. Surfactin (SF), synthesized by *B. subtilis*, is a naturally derived surfactant (biosurfactant). It is a cyclic lipopeptide with high surface activity, and at the same time, it is biodegradable. Surfactin is non-toxic to healthy cells, including the keratinocytes HaCaT cell line and has anticancer properties [15]. The ideal solution is to use two simultaneous nanosystems-allowing to deliver the active substances into the deeper layers of the skin and increasing skin hydration.

This research aimed to incorporate two types of nanosystems–levan nanoparticles (NP) and nanoemulsion (NE) stabilized by surfactin–in the three topical formulations. Model formulations of tonic, gel, and cream were chosen for incorporation during the production process in matrix single and hybrid double dispersion. Firstly, preservatives have been tested, and their effect on the stability of the systems has been examined. Additionally, the results have been confirmed by the DLS method. Change in the size of drops, the zeta potential of the dispersion, and polydispersity were measured. All formulations were compared to each other in antiradical properties by radical scavenging EPR technique, analyzing the synergistic effect of nanosystems with matrices. The incorporation of NP and NE has been confirmed by imagining by TEM. In the end, the skin condition was assessed using the analyzer: structure, hydration, pore size, depth of wrinkles, degree of discoloration, and the properties were confirmed by ten women, aged 42– 54 years, using the preparation.

## 2. Results and Discussion

### 2.1. Preservation, Evaluation of Microbial Protection, and Stability of Nanosystems

The main task of the preservative is to keep the product-during storage and use-in the same microbiological purity in which it was produced. The addition of preservatives also prevents the formation of microbial metabolic products, which can cause skin irritation. Preparations of products, especially those that contain a large amount of water, are prone to the growth of microorganisms [16]. As a result of microbiological contamination, they lose their physicochemical properties and utility values. Thanks to preservatives, it is possible to maintain microbiological purity during use and storage. A preservative introduced into a system should meet several criteria: it should be active at a low concentration and in a wide pH range and be effective against a wide range of microorganisms [17]. It mustn’t be toxic, irritating, or allergenic. It must be chemically inert, resistant to light, oxygen, and temperature changes. Therefore, the selection of a preservative for a particular system is a challenging task.

Our work aims to incorporate nanoemulsion systems and nanoparticles of levan into cosmetic matrices. These systems, dispersed in water, are introduced into more complex matrices in which the main component is water. Therefore, the first step was to select an appropriate preservative for nanosystems. In the case of preservation of nanocarriers, antimicrobial agents should not affect their stability as well. Preservatives are known for causing physical instability of disperse systems such as aggregation, coalescence [18]. The first stage in this part of the work was to select an appropriate preservative for the developed nanoemulsion. Nanoemulsions are more complex and are less stable systems than nanoparticles. Hence, the preservative must not disturb the stability of the nanoemulsion.

Pivotal role during selection was driven by their chemical performance: hydrophilic (e.g., propylene glycol, sodium levulinate, sodium benzoate), and hydrophobic (e.g., glyceryl caprylate, glyceryl undecylenate) (Table 1). Additionally, an emerging class of multifunctional-ingredients that besides antimicrobial properties, possess skin-caring properties, thus do not have to be declared as preservatives such as pentylene glycol, which was already used in nanostructured carriers [19]. The concentration of applied preservatives was at the highest dose allowed since, at this time point, it is difficult to predict for which products each nanocarrier will be used, or the kind of microbial exposure during the shelf life of the final products. Adequate pH is an important issue, as it can destabilize the carrier, in particular nanoemulsions. For this reason, a pH range of preservative had to strongly correlate with the actual pH of the nanocarrier. The influence of 26 preservatives, in three different temperature ranges, was tested on the stability of nanoemulsion. Since nanodispersions are vulnerable to chemical destruction, it was important to conduct possibly the widest range of inspections. A group of preservatives containing benzyl alcohol (C, J, M, P), caused changes in the visual appearance of the samples, due to sedimentation or phase separation. Similar results were obtained when preservation systems for nanostructured lipid carriers (NLC) were evaluated [18]. Other preservatives such as B, D, E, H, I, O, S, AC caused destabilization of the systems by sedimentation or phase separation. Preservatives showing signs of aggregation, precipitation, or sedimentation were rejected when visually tested.

After selecting the appropriate preservatives for the nanoemulsions, the next step was to check whether any of the typed preservatives would also be stable and have antimicrobial effect on the solution of levan nanoparticles. Since levan nanoparticles are considered to be stable systems, stability tests were narrowed regarding temperature, as well as the number of tested preservation systems (Table 1). Among 12 selected preservatives, four did not influence the stability of levan nanoparticles while inspected visually (Z, AA, AB, AD). When those systems were inspected regarding the presence of microorganisms, the only preservative consisting of glycerin and propylene glycol was shown to be effective (AA). The pH value of the unpreserved levan nanoparticles dropped significantly during observation time, as well as in the case of preservatives that were not suppressing growth of microorganisms. 

In nanosystems, it is not always possible to see the symptoms of aging, instability, or aggregation. Hence, an important element is the DLS analysis. It allows determination of not only the size of a given carrier but also their polydispersity and the Zeta potential. Analysis of those parameters may indicate their stability. The most desirable PdI value is around 0.2. However, in the case of the Zeta potential high values indicate high stability of the carrier, which is important during the manufacturing processes. 

Samples of nanoemulsions after preservation with previously selected antimicrobial agents for further tests were tested by means of DLS analysis (Table 2). Shortly after preservation, most of the samples had a small particle size below 220 nm. In the case of glycerin, when added to the nanoemulsion, the size of the carrier increased (preservative Z, AA). The same observation was made for preservative N. Reduction of carrier size was observed when antimicrobials composed of levulinate salts were added (K, L). The decrease in the carrier size was obtained when 1.2 hexanediol was added to the system (R, AE), propylene glycol (W) as well as phenylpropanol combined with pentylene glycol (F). Very little changes in the particle size were observed when pentylene glycol on its own (AB) or combined with citrus extracts (A), butylene glycol (AD), or salts of benzoic acids and sorbic acid were added (G). 

There were also changes in the polydispersity of these samples, but they are still very optimal and close to 0.200. Changes resulting from the stabilization of the systems were observed with regard to the Zeta potential.

Preservation systems that gave satisfactory results after DLS measurements were subjected to preservation efficacy test, so-called challenge test. There were 12 antimicrobial agents tested for their protection efficiency (Table 1). Nanoemulsions preserved with promising antimicrobials were challenged with two microbial strains *E. coli*, *S. aureus,* and yeast *C. albicans*. The initial number of microbial cells in the inoculum and 1g of the tested formulation is given in Table S1. Prepared microbial suspensions meet criteria set in normative document ISO 11930. Validation of neutralizer efficiency was performed, and according to the collected data diluent used (D/E broth), neutralized preservatives without inhibition of microbial growth (Table S2). 

Pentylene glycol, by itself or combined either with phenylpropanol or with citrus extracts meet the criteria given in Table S3 (preservative symbol A, AB, and F). Nanosystems preserved with 1.2-hexanediol gave a satisfactory reduction of microbial growth (R). A synergistic effect was observed when 1.2-hexanediol was added together with butylene glycol (AE), the former did not present antimicrobial properties when added alone. Satisfactory results were obtained for a mixture of levulinic acid and its sodium salt (N). However, these preservatives are considered to be mild, and may not withstand harsh production conditions (Tables S4 and S5). 

Levan nanocarriers are prone to microbial deterioration since they are of bacterial origin. Preliminary tests, consisting of a simple microbial purity check, showed, that out of 13 tested preservatives, only one was effective to suppress bacterial growth, and further was examined for its preservation efficacy. Antimicrobial agent composed of glycerin and propylene glycol (AA) turned out to be effective during a challenge test (Tables S4 and S5) and the next part of the research. 

### 2.2. Incorporation of Nanosystems

#### 2.2.1. Into the Model Matrix

The ingredients used in cosmetic products depend greatly on the type of formulation (cream, gel, emulsion, lotion, etc). However, the principal raw materials used to manufacture cosmetics are water, oily materials, fats, and wax, surfactants, humectants, thickening agents, antioxidants, preservatives, coloring agents, along with vitamins, as well as active agents. Cosmetic emulsions based on vegetable oils are the subject of many studies reported in the literature. They are preferred instead of mineral oils, due to their characteristics: biocompatibility and biodegradability, effectiveness in protecting the skin against solar radiation, inflammation, insect attack, microorganisms, and viruses [20]. Moreover, in comparison to mineral oils, vegetable oils exhibit low viscosity and molecular weight, which makes them less occlusive [21]. Therefore, research cream formulation (emulsion-EM) was created based on vegetable oils. An emulsion is a mixture of two immiscible liquids with different polarities where one liquid is dispersed as often deformed droplets into the other phase. The emulsions used in cosmetic formulations are usually based on water (W)-oils mixtures (O). There are two main types of W–O systems: one of them consists of water droplets dispersed in an oil phase (called water-in-oil (W/O) emulsion); the other one is represented by dispersed droplets of oil in the aqueous phase (called oil-in-water (O/W) emulsion). As a general rule, the type of emulsion depends primarily on the emulsifier type (i.e., its HLB number) used in the formulation. O/W emulsions are commonly used as water-washable drug bases and for general cosmetic purposes while W/O emulsions are widely used as emollients and for dry skin, treatment [22,23]. Important to note that when applied topically, constituents of the formulation may act synergistically by several mechanisms: promoting skin barrier homeostasis; antioxidative activities; anti-inflammatory properties; anti-microbial properties; promoting wound healing; and anti-carcinogenic properties [24]. This is the reason for using in our study mixtures of all the above components. The developed base formulation is stabilized by nonionic emulsifiers that create hydrogen bonds and spatial obstacles that prevent dispersed droplets from approaching one another. The introduction of an ionic emulsifier in nanoemulsion creates an electrically charged film on the surfaces of the dispersed phase droplets; this effect repels the droplets from one another, therefore the product is stable.

The cream’s composition has been developed exclusively based on natural ingredients. The cream has been specially designed in such a way as to take into account all aspects, both technological, application, and safety-related. The prepared recipe is an O/W emulsion. When developing a recipe, the ingredient necessary to create an emulsion is an emulsifier. The emulsifier polyglyceryl-3 picitrate/stearate (TEGO^®^ Care PSC 3-Evonik) was used. It is a non-ionic emulsifier, obtained in the process of esterification of polyglycerol-3 with stearic acid and citric acid. This emulsifier has a good emulsifying effect and good skin tolerance. Polyglyceryl-3 dicitrate/stearate in combination with consistency-forming ingredients, such as glyceryl stearate and cetearyl alcohol, creates emulsions building lamellar structures. This emulsifier is adapted to emulsions, in which it works synergistically with the natural organic acids used.

Emollients are an essential element of the cream. They show regenerative, and moisturizing properties. Emollients help replenish epidermal lipids. They include, among other ceramides, triglycerides, linoleic and linic acid, and cholesterol. Additionally, they contain natural vegetable oils. Such a set of ingredients allow the rebuilding of the damaged lipid layer of the epidermis. Occlusive substances are another component of emollients. Producers play this role, among other petroleum jellies, paraffin, fatty acids, and phospholipids. These substances allow you to create a thin protective layer on the epidermis, which will prevent water loss from the deeper parts of the skin.

To provide the emulsion with appropriate sensory properties and better distribution, the following emollients were used, such as *Butyrospermum parkii* (Shea) butter, squalane, sesame oil, oleic/linoleic/linolenic polyglyceride, caprylic/capric triglyceride, octyldodecanol emollient, triheptanoin. They are all characterized by great skincare properties.

As for substances that increase and control the condition of skin hydration, propylene glycol, and glycerin were used. Thanks to the ability to penetrate the stratum corneum, glycerin acts as a promoter, making it easier for active substances to penetrate deep into the skin. It homogenizes cosmetic ingredients and is also a humectant with gentle preservation properties. Propylene glycol, vegetable glycol, is an excellent carrier of active substances and a solvent for acids and plant extracts. Thanks to a small molecule, it easily penetrates deep into the epidermis, improving its hydration level.

The cream also contains two ingredients from the group of active substances-tocopherol and *Punica Granatum* fruit extract. Tocopherol is an antioxidant. It protects cosmetic products, including oils, against oxidation. The *Punica Granatum* fruit extract helps protect the skin from damage by free UV radicals. It contains large amounts of polyphenols, including anthocyanins, gallotanin, and ellagitanin with strong antioxidant and astringent properties. In addition, substances increasing the viscosity of the entire formulation were used, such as microcrystalline cellulose and xanthan gum, as well as the preservatives benzyl alcohol, benzoic acid, and dehydroacetic acid.

The serum components have been selected in a way to facilitate the penetration of active substances. Permeation promoters are a mixture of natural glycols (propylene glycol and pentylene glycol). These substances also improve the condition of the skin.

One of the ingredients of the tonic is betaine. It has a protective effect on cell membranes and has a soothing and moisturizing effect. Another ingredient is sodium L-pyroglutamate-it is present in the skin as a component of NMF (Natural Moisturizing Factor), which allows water to be retained in the stratum corneum. It is one of the most effective moisturizing factors. It permanently moisturizes the epidermis and deeper layers of the skin softening it and toning. Another active ingredient is aloe vera juice, which has a strong moisturizing, softening, and antioxidant effect. It also has anti-inflammatory properties and speeds up wound healing.

#### 2.2.2. Compatibility of Nanosystems with Matrix

**Figure 1:**
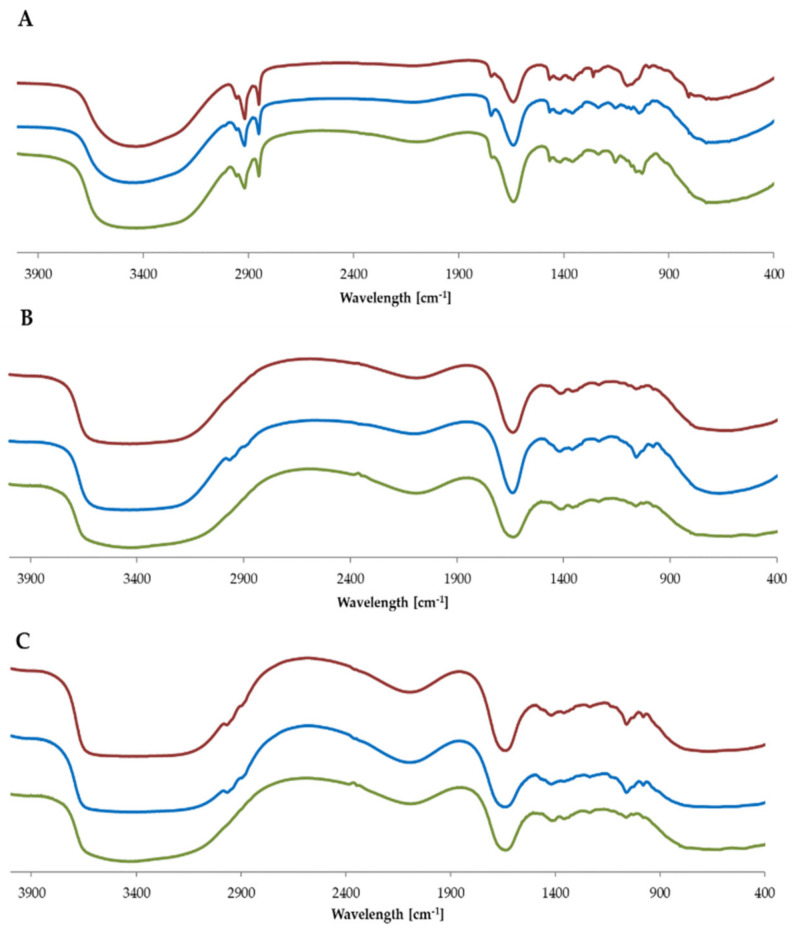
IR spectra of (**A**) cream, (**B**) tonic and (**C**) serum. In red-empty formulation, in blue-with levan nanoparticles, in green-with nanoemulsion.

The IR analysis shows that there are no spectral differences between empty formulations and formulations enriched with levan nanoparticles or nanoemulsion (Figure 1). This observation proves that there are not any side processes after adding ingredients to the matrix and the matrix remains stable [25]. For all three types of formulation, the most intensive signals are connected with water molecule oscillation (~3000–3700 cm^−1^) and the presence of alcohols like glycerol, propylene glycol (hydroxyl group oscillations ~1500 cm^−1^). In the case of cream, there are also characteristic bands associated with aliphatic groups from fatty acids (~2900 cm^−1^) [26]. 

#### 2.2.3. Imaging of Implementation

**Figure 2:**
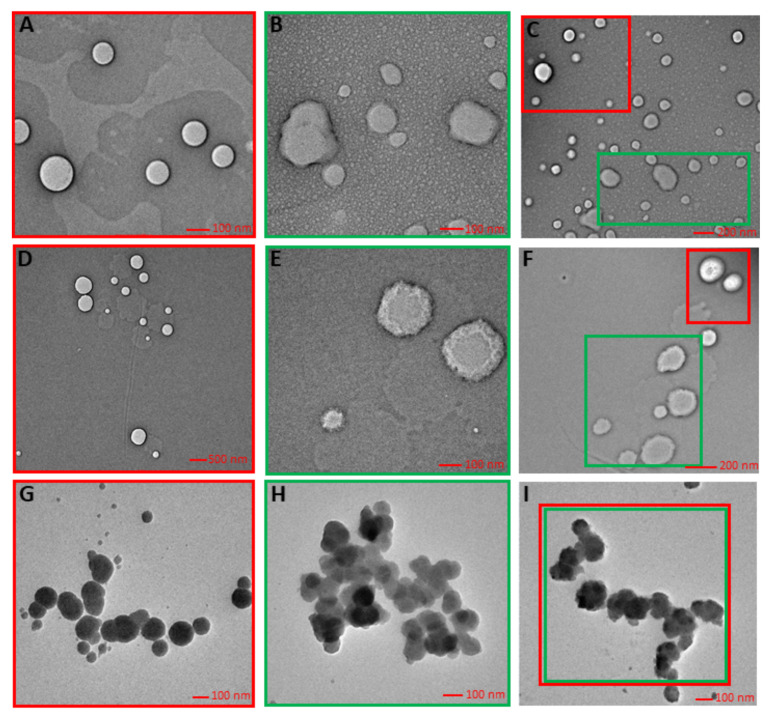
Implementation of nanosystems in matrix: (**A**) nanoemulsion; (**B**) levan nanoparticles; (**C**) hybrid double dispersion-in cream; (**D**) nanoemulsion; (**E**) levan nanoparticles; (**F**) hybrid double dispersion-in serum; (**G**) nanoemulsion; (**H**) levan nanoparticles; (**I**) hybrid double dispersion-in tonic.

Transmission electron microscopy (TEM) is the rapid, powerful, and relatively non-invasive visual technique to obtain information about the mean size and the actual surface and morphology characteristics of the prepared and incorporated nanosystems in the matrix. Imaging was performed for all three prototype formulations with the incorporation of two types of systems (Figure 2).

The TEM photographs revealed that nanoemulsion was of spherical morphology having diameter range from 220 nm to 300 nm, and levan nanoparticles show a deformation from a spherical form, appearing in irregular shapes independently of the matrix used and with particle size 130–260 nm. The diameter of NE and NP-levan obtained using DLS (Table 2) and TEM were in reasonable agreement with each other and the difference came from the different sample preparation processes and different principles.

### 2.3. Antiradical Properties of Incorporated Nanosystems in the Matrix

Due to the capability to oxidize lipids, proteins, and nucleic acids, free radicals are one of the skin aging factors. Hence, radical scavenging is an essential property of cosmetic formulations [27]. Moreover, it was investigated that some antioxidant compounds can exhibit also antimicrobial agents [28]. Therefore, the antiradical effect of levan nanoparticles and nanoemulsion in the prototype of cosmetic formulations as cream, tonic, and serum was examined. The most accessible and most common method to evaluate the antiradical properties is scavenging of DPPH by UV-Vis spectroscopy [29] but can only be used for clear samples. In the case of cosmetic preparations, with a consistency of creams, the turbidity of the system was difficult, so it was decided to use EPR technique [30]. DPPH radical is used as a radical molecule to evaluate the free radical scavenging ability of the compounds. The DPPH radical is characterized by a strong EPR signal intensity, and it can accept an electron or hydrogen atom to become a stable diamagnetic molecule. The loss of the DPPH EPR signal intensity in the presence of antioxidants is directly proportional to the concentration (or number) of electrons (protons) accepted. The EPR signal intensity was stable but it was rapidly reduced after the addition of the test compound in a concentration-dependent manner. 

**Figure 3:**
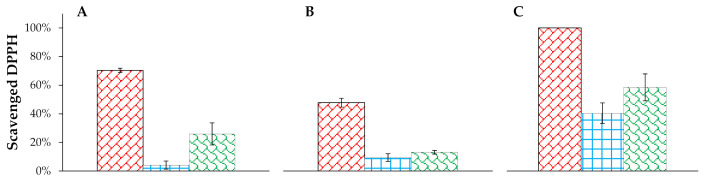
Antiradical effect of ingredients in three different types of formulations: **A**—levan nanoparticles, **B**—nanoemulsion, **C**—the linkage of levan nanoparticles and nanoemulsion. 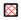
—cream; 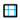
—tonic; 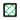
—serum.

At first, the antiradical potential of levan nanoparticles, nanoemulsion, and their linkage in three different formulations (cream, tonic, serum) were investigated. The samples were measured 30 min after adding the DPPH solution. These results showed us that in all formulations free radical scavenging effect is observed (Figure 3). Empty formulations were investigated simultaneously with enriched ones to examine if there is any antiradical effect associated with the matrix. Our results showed that there is no change in radical signals’ intensities-differences between samples measured after 5 and 30 min were smaller than their standard deviations. Therefore, we concluded that in the case of empty cream, serum and tonic there is no antiradical effect.

**Figure 4:**
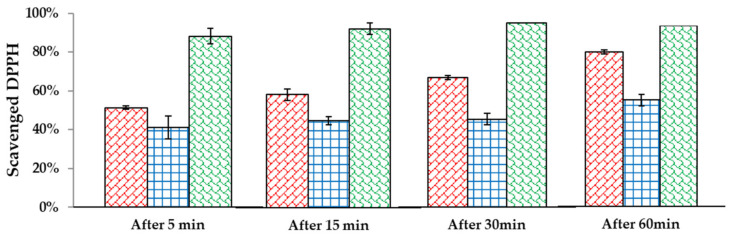
Time-dependent radical scavenging in cream. 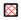
—levan nanoparticles; 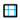
—nanoemulsion; 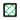
—linkage.

In the case of tonic and serum, the scavenging of free radicals was moderate, but the cream showed much greater properties in removing them. Moreover, in all three formulations, a synergy of nanoemulsion and levan nanoparticles linkage was observed. Such an effect was described also in other systems [31]. Due to the satisfying effect of the cream, radical scavenging in time was determined (measuring EPR signal after 5, 15, 30, and 60 min after adding DPPH). Figure 4 shows the differences in the percentage of scavenged DPPH in time. According to the results, a cream containing both levan nanoparticles and nanoemulsion is capable of terminating 88% of DPPH after 5 min and 100% after 30 min. It means that 100 µL of our cream formulation diluted 5 times in water can neutralize 0.152 µmol of radical in half an hour. Similar research was done with a formulation containing *Sterculia populifolia* extract as an antioxidant [32]. Its authors showed that a concentration of 100 µg/mL of this extract can neutralize 50% of radicals in 3 mL of 0.004% (w/w) solution of DPPH, which gives 0.152 µmol. Moreover, 100 µL of 0.015 mM solution of ascorbic acid can terminate 0.2 µmol of DPPH [33], hence it can be concluded that the tested formulations have comparable antioxidant properties.

### 2.4. Application Study and Skin Conditioning-In Vivo

**Figure 5:**
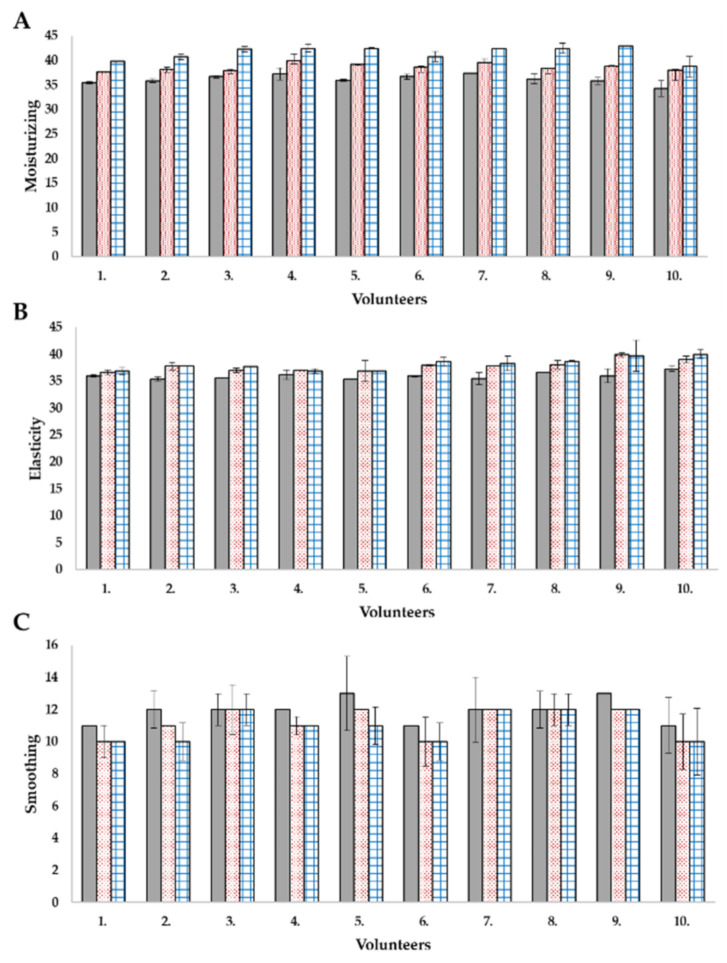
In vivo application tests: (**A**) moisturizing, (**B**) elasticity, (**C**) smoothing. 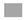
—before testing, 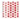
—after series of applications of the base cream, 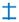
—after series of applications of the cream with nanosystems.

Cosmetic preparations for application on the skin (such as creams, serums, tonics, etc.) are designed to protect the skin, remove its small defects, such as by smoothing wrinkles or whitening discoloration. However, for these processes to take place, the active substances contained in the cosmetic must penetrate the deeper layers of the skin. The use of nanosystems enables the transport of active substances and may support skin repair processes. Application tests carried out on volunteers using non-invasive methods are one of the possibilities of testing preparations before their commercialization [34]. In this study, the influence of prepared base cream and cream with nanosystems were evaluated. The effectiveness of the product is confirmed with positive results in at least 50% of the volunteers participating in the study. The moisturizing, elasticity, smoothing, depth, and volume of wrinkles were measured in a non-invasive way. The cream containing nanosystems increased skin hydration by 14.9%, while the base cream alone increased the hydration by 6.6% (Figure 5A).

In the case of elasticity, both creams had a similar effect, but the one containing levan and nanoemulsion was 1.1% more effective (for the base cream, an increase of 5%) (Figure 5B). The respondents paid attention to the smoothing of the skin, which was also shown by apparatus tests-the cream with nano-systems smoothed the skin by 1.7% better than the base cream itself (Figure 5C). 

**Figure 6:**
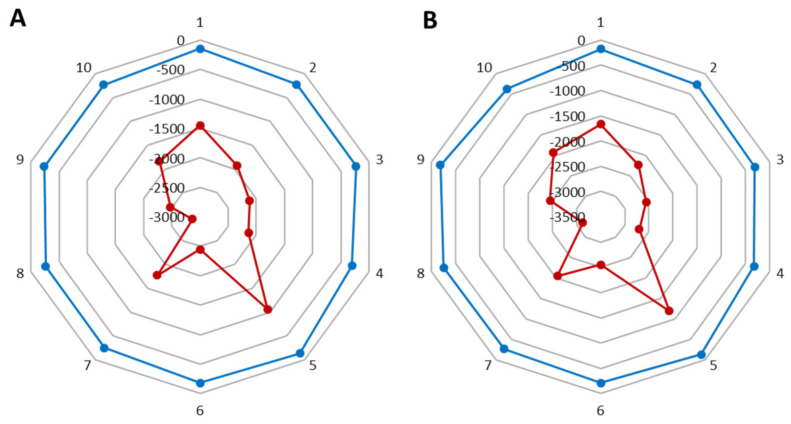
Differences in the volume of the wrinkles (red) and its surface (blue) (**A**) after a base cream and (**B**) cream with the nanosystems.

**Figure 7:**
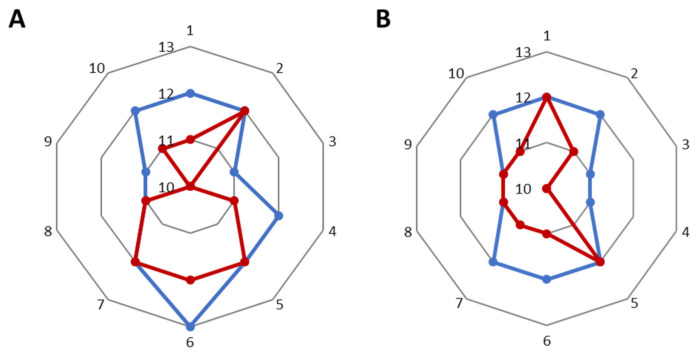
The depth of the wrinkles before application (blue) and after application (red) (**A**) of base cream, (**B**) of cream with nanosystems.

After using the cream with levan and the formulation, the volume and area of wrinkles decreased by 2.3 and 3.2%, respectively (where for the base cream the parameter improved by 2.1 and 2.8%) (Figure 6). In the case of wrinkle depth for the base cream, there was a 6.8% decrease (Figure 7A), while for the cream with nanosystems this decrease was smaller and amounted to 5.2% (Figure 7B). All results are given in Tables in SM (Tables S6–S10).

Based on skin analyzes carried out with the use of NatiV3, the effect of the cream, serum, and tonic with two nanosystems on vascular lesions and discoloration was determined. Comparing the base cream with the range of products with nano-systems, their effect on the vascular lesions was negligible. The values for both tested versions of skincare are similar to each other, and additionally, they are burdened with quite a large standard deviation. For the base cream, the size of the vials was 1.87 ± 1.28 mm^2^, and after 28 days of use, 1.41 ± 1.28 mm^2^. For the products with nanosystems, this value was 2.27 ± 0.95 mm^2^ before application, and after 28 days of daily applications, 1.37 ± 1.16 mm^2^. In the case of discoloration, it was found that the base cream did not reduce the discoloration on the skin after 28 days of use, unlike the cosmetics with nano-systems. Initially, the discoloration was 10.91 ± 3.20% and 10.88 ± 0.72%, and after 28 days of use, it was 11.29 ± 0.79 and 10.42 ± 0.58%, respectively for the base cream and cosmetics with nano-systems. Sample of changes before applying the formulation and after 28 days of use in Supplementary Materials (Figure S1).

The analysis of a given preparation is difficult to interpret. The skin is not a homogeneous tissue and it is characterized by high variability in parameters, even within one examined person. The skin’s reaction to a given cosmetic also depends on the state of health, environmental conditions, etc. The developed preparation with a double nanosystem is only at the initial stage of research. Further work is underway to improve it. The studies conducted so far have shown that their use in cosmetic preparations has a positive effect on the condition of the skin. The use of polymers in the production of cosmetics is very common. Their addition has a great influence on the consistency of the preparations, increasing their viscosity and ensuring greater stability of the emulsion. The addition of more than one polymer can lead to faster coalescence or isolation of the emulsion droplets. The use of polymers in cosmetics also affects their care properties. They can retain water in their structures, thus increasing skin hydration. The polymers can also form a scaffold for other essential ingredients, e.g., ions, or form a coating around sensitive active substances. The use of bacterial cellulose in cosmetics include facial masks, facial scrub, personal cleansing formulations, and contact lenses has been reported so far [35]. Another used polymer is chitosan and its derivatives. The encapsulation of, for example, essential oils or polyphenols with strong antioxidant properties in chitosan protects their action, as they are usually very sensitive to external factors [36]. However, the use of at least two polymers in a cosmetic preparation may result in the creation of new materials based on the molecular interaction between polymer molecules [37]. One of the greatest advantages of using nanoemulsions in cosmetics is dissolving large quantities of hydrophobic compounds and the ability to protect drugs from degradation [38]. In addition to the interest in nanoemulsions due to their unique consistency, other benefits of nanoemulsions have been appreciated in many cosmetic applications, especially skincare for solubilizing and delivering active ingredients [39]. Enclosing the active substances in the nanoemulsion enables their transport through the stratum corneum to the deeper layers of the skin [7]. So far, various nanoemulsions have been tested for their cosmetic use. Their use is to moisturize the skin [40,41], lighten discoloration [42], or reduce vascular lesions [7]. The formulation presented in this study has the potential to improve the condition of the skin and slow down the aging processes.

## 3. Materials and Methods

### 3.1. Levan Nanoparticle Preparation

Nanoparticles of levan were prepared with a precipitated and lyophilized polymer. An appropriate amount of levan was taken to prepare a 5% solution in distilled water. It was stirred till it dissolved. Then the solution of levan nanoparticles was incorporated into the cream. Levan was obtained from microbial fermentation with *Bacillus subtilis* natto KB1, described below. Levan standard was purchased in Sigma-Aldrich (Poznan, Poland). All medium components were purchased from BioShop LabEmpire (Rzeszow, Poland). Ethanol and minerals were purchased in Chempur (Chempur, Poland). All other reagents were of analytical grade and used as provided. Water used for all experiments was doubly distilled and purified through a Milli-Q water purification system (Millipore, Bedford, MA, USA).

The culture of *Bacillus subtilis* natto KB1 was grown at 37 °C for 24 h with continuous shaking in a 5 L bioreactor. After 24 h, the culture was stopped and centrifuged to separate the bacterial biomass. Levan was separated from the supernatant as described before [11].

### 3.2. Nanoemulsion Preparation

Nanoemulsion concentrate was prepared as follows: 50% of sodium surfactin powder, 30% of 2-(2 ethoxyethoxy) ethanol (trade name Transcutol HP; Gattefossé SAS, Saint-Priest, France) and 20% of ascorbyl tetraisopalmitate (trade name Nikkol VC-IP, Nicco Chemicals Co. Ltd., Tokio, Japan) were subjected to ultrasound for 20 min, at 50 °C, as previously described [7]. Concentrates were organoleptically inspected for the absence of any thickenings. In case there were none, the obtained pre-concentrate was diluted in a glass beaker, 50 mg to 10 mL of water at temperature 37 °C, and further stirred on a magnetic stirring plate. 

Surfactin was obtained from microbial fermentation with *B. subtilis* natto KB1, as described before [7,43]. All medium components were purchased from BioShop LabEmpire (Rzeszow, Poland). All solvents for chromatographic separations were of HPLC and/or MS grade for HPLC and MS analyses, respectively. Trifluoracetic acid (TFA) and surfactin standards were purchased from Sigma Aldrich.

### 3.3. Other Components

Following ingredients were obtained as a gift: Dermosoft PEA, Dermasoft GMCY, Dermosoft 1388 Eco (Evonik, Essen, Germany), Activonol BG (Activon, Suwon City, Korea), Stabil Zero, Kem Nat, Kem Nat Beta, Kem DH (Akema, Coriano, Italy), Liqupar ME (Ashland, Wilmington, NC, USA), Cosphagard Elbe, Cosphagard Pol, Cosphaderm LA-T, Cosphaderm TOM, Cosphaderm Hexiol, Cosphaderm Octiol, Cosphaderm Sodium LAAS (Cosphatec, Hamburg, Germany), InBioLev, InBioSur (InventionBio, Bydgoszcz, Poland), Geogard ECT, Geogard Ultra (Lonza, Basel, Switzerland), E-Leen Green OR, MinaSolve Green A, A-Leen Aroma-3 (MinaSolve, Mont-Saint-Guibert, Belgium), Euxyl PE 9010, Euxyl K340, Euxyl K320 (Schülke, Warsaw, Poland), Sharomix 703 (Donauchem, Rokietnica, Poland). Other raw materials were purchased, Ajidew NL-50 (Ajinomoto, Tokyo, Japan), Viamerine 2500 (Aldivia, Saint-Genis-Laval, France), Aloe Vera SDP 200X (Ashland, Wilmington, USA), Myritol 312, Eutanol G, Cetiol SB 45, Lanette O, Cutine GMSV, Plantacare 810 (BASF, Ludwigshafen, Germany), Propylene Glycol (Brenntag, Kędzierzyn-Koźle, Poland), Cosphaderm 1,3 -Propanediol, Cosphaderm X34 (Cosphatec, Hamburg, Germany), Glycerin (CQM Masso, Warsaw, Poland), Fruitliquid Pomegranate (Crodarom, Chanac, France), Dermofeel PA 12, Tego Natural Betaine, Tego Care PSC-3, Dermofeel Toco 70 non, Dermofeel PA-12 (Evonik, Essen, Germany), Transcutol HP (Gattefossé SAS, Saint-Priest, France), Sesame Oil (Henry Lamotte, Bremen, Germany), Miglyol T-C7 (IOI Oleochemical, Putrajaya, Malaysia.), A-Leen 5 (Minasolve, Mont-Saint-Guibert, Belgium), Nikkol VC-IP (Nicco Chemicals, Tokyo, Japan), Vivapur CS 032 XV (Rettenmaier, Rosenberg, Germany), Natural Parfume (Robertet, Grasse, France), Euxyl K903, Euxyl K903 (Schülke, Norderstedt, Germany), Sharomix 713 (Donauchem Polska, Rokietnica, Poland), Phytosqualan (Sophim, Peyruis, France).

### 3.4. Influence of Conservation Systems on Stability 

#### 3.4.1. Preservation Efficiency

Obtained nanoemulsions were subjected to various preservation strategies. Homogenization of nanoemulsions was followed by the addition of antimicrobial agents according to Table 3, and tubes were kept in three different conditions: refrigerator at 4 °C, an incubator at 37 °C, and climate chamber set at 50 °C, 75% humidity, light 7500 lux/h, 1.1 W/m^2^ (UVA). After every month of incubation, microbiological tests for the presence or absence of mesophilic microorganisms as well as for yeast and molds were conducted as described in SM1. The obtained results were assessed according to criteria given in the normative document ISO 17516 (Table S11). 

#### 3.4.2. Evaluation of Microbial Protection

The preservation efficiency test was performed according to the method described in the normative document ISO 11930 (SM2). Nanoemulsions have been subjected to stress tests to assess the effectiveness of preservation [44]. Microorganisms used in the test were: *Escherichia coli* (ATCC 8739), *Pseudomonas aeruginosa* (ATCC 9027), *Staphylococcus aureus* (ATCC 6538). The obtained results were assessed according to the criteria given in Table S3. The composition of the media used in the experiment are given in Table S12, incubation conditions are given in SM2. Bacterial suspensions were prepared as mentioned in SM4. Enumeration of microbial cells in the inoculum was performed using the pour plate method (SM3). The initial number of viable microorganisms in the inoculum and 1g of the tested formulation is listed in Table S1.

#### 3.4.3. Stability of Nanosystems

The integrity of nanocarriers with preservatives was inspected organoleptically right after preparation (D0), after 30 (D30), and 90 (D90) days of incubation at three temperature conditions. pH was measured after preparation (D0) of all prepared nanocarriers and was continued in case of visual integrity. DLS measurements were taken in case of organoleptic acceptance to overcome the problem of unstable formulations, nanoemulsions were subjected to time-dependent size (D_H_) and Z-potential measurements after preparation (D0) and 90 days (D90) of storage as well as dispersion stability test [7]. 

### 3.5. Incorporation of Nanosystems into the Model Matrix

The formulation prototypes (new design) have been developed for this work. All the ingredients were carefully selected and used for the formulations according to the rules provided by The European Cosmetics Legislation [45]. The main raw materials used to obtain cosmetics are water, oily materials (oils, fats), humectants, antioxidants, preservatives, active agents, fragrances. The compositions of the prepared cosmetic emulsions are shown in Table 4, Table 5 and Table 6. The classic type emulsion oil in water (O/W), was chosen as the cosmetic matrix model, where the external phase is water and the internal (dispersed phase) is oil (Table 4). Whereas to test the compatibility of nanosystems with water formulations, recipes for serum (Table 5) and face tonic (Table 6) were prepared. 

Preparation:

Phases A and B were heated separately to a temperature of 70–75 °C, which was followed by homogenization. Raw materials from phase C were added into the water at 40–45 °C and mechanically stirred for about 1 h. Phases A and B were poured into one vessel and subsequently phase C was added. Such a prepared mixture was homogenized. Afterward, the emulsion was cooled down by a mechanical stirrer at low speed. Phase D was added and pH was set to the value of 5.5 with citric acid. Previously prepared nanosystems (phase E) were mixed into the emulsion at the final step (Table 4). 

#### 3.5.1. Images of Incorporated Nanosystems in Different Matrix

The transmission electron microscopy (TEM) measurements were performed to measure the morphology and size distribution of lipid nanocarriers. Images were taken using an FEI Tecnai G2 20 XTWIN electron microscope (FEI, Hillsboro, OR, USA). The size distribution of the nanocarriers for each sample was determined by counting the size of approximately 250 nanocarriers from several TEM images obtained from different parts of the TEM grids. A few drops of the diluted suspension were placed on the grid and stained with 2% uranyl acetate, and then the image was captured. The size distribution plots were fitted by using Gauss curve approximation.

#### 3.5.2. IR Analysis of Nanosystems in the Cosmetic Matrix 

Functional groups and chemical bonds of the obtained cosmetic products were determined using Fourier-transform infrared (FT-IR) spectroscopy on Spectrometer Bruker Vertex 70 FT-IR (Bruker Optics, Ettlingen, Germany). The sample was prepared as a thin film. All of the samples were scanned over a wavelength range of 4000–400 cm^−1^.

### 3.6. Antiradical Properties of Incorporated Nanosystems in the Matrix

Antiradical properties of self-assembly nanosystems incorporation in the different matrix were studied by radical scavenging activity using DPPH by electron paramagnetic resonance. A stable radical solution in ethanol was stirred in dark for 2 h to obtain a homogeneous solution. Next, the tested systems were prepared as follows: 75 μL of radical and 100 μL of the sample (5x diluted in water) were mixed. The capillary tube with the sample was sealed and placed inside a standard EPR quartz tube, then placed in the resonant cavity. Measurements were carried out at room temperature. Data were reported as the average of three measurements. The EPR spectra were recorded using a Bruker Elexsys 500 spectrometer operating at the X-band frequency (~9.7 GHz). A microwave power of 1 mW, modulation amplitude of 1 G, a sweep width of 200 G were adopted. An analysis of the EPR spectra was carried out using the WinEPR software package, version 1.26b (Bruker WinEPR GmbH, Rheinstetten, Germany). The double integral of the signal was evaluated as representative of the free radical concentration. The field under the absorption curve is proportional to the number of stable radicals remaining in the sample. The percent value of scavenged DPPH was calculated with the following formula:

$$Scavenged\;DPPH=\frac{I\;standard-I\;sample}{I\;standard}*100\%$$

*I*_sample_—integrity of signal measured in the sample

*I*_standard_—integrity of signal measured in the sample

### 3.7. Conditioning Skin In Vivo 

For in vivo evaluation of vascular lesions, discoloration, NatiV3 Analyzer (Beauty of Science, Poland) was used [7]. The measurements were analyzed based on the manufacturer’s database (more than 2500 studies of people between 18 and 90 years old, both women and men) [46]. Prepared cosmetic formulations C1, C2, C3, C4 of cream separately. In the next research, the effect of using the product group consisting of tonic, serum, and cream was tested. T4, S4, and C4 were applied to the skin and compared with part of the face with C4 only. The measurements were carried out before the application of the formulations and at 28 days after application.

### 3.8. Application Tests In Vivo

The test was performed on 10 women, age 42–54 years. Measurements were taken on healthy skin, without irritation signs. Volunteers were advised not to use any antihistamines or pharmacological drugs, which could interfere with the results. Both formulations were applied daily, one without the addition of NP levan, and NE was applied on the left part of the face, neck, and neckline, while the formula with NP levan and NE on the right part of the body. The measurements were taken before the application of formulations, and after four weeks of application. All measurements were carried out in a room with a temperature of 20 °C and relative humidity of 50 ± 10%. 

Performed methods were designed in accordance with Regulation (EC) No 1223/2009 of the European Parliament and of the Council of 30 November 2009 on cosmetic products; Cosmetics Europe-The Personal Care Association Guidelines Product Test Guidelines for the Assessment of Human Skin Compatibility 1997; Cosmetics Europe-The Personal Care Association Guidelines for the Evaluation of the Efficacy of Cosmetic Products 2008 and the laboratory procedure No. PB-19 ed. I from November 10, 2017 “Cosmetic products. Apparatus application research”.

For the assessment of skin hydration, a Multi Probe Adapter System was used (Courage+Khazaka Electronics, Cologne, Germany), which is connected to a probe used for the determination of the skin surface hydration-Corneometer^®^ CM 825, for firmness/elasticity–Cutometer MPA580, smoothening effect–Visioscan VC98, reduction of wrinkles–Visioline VL650. All the measurements were performed in triplicate (mean ± SD, *n* = 3). The obtained results are presented as absolute numbers.

### 3.9. Statistical Analysis

All data are expressed as the mean value ± standard deviation of three measurements. Statistical analyses were accomplished using an online one-way analysis of variance with the post hoc Tukey honest significant difference calculator. A value of *p* < 0.05 was considered statistically significant.

## 4. Conclusions

Antimicrobial agents were tested on the nanoemulsion and levan nanocarrier stability. For nanoemulsions pentylene glycol, 1.2-hexanediol and butylene glycol proved to have a preservation effect. In the case of levan nanocarriers, only glycerin combined with propylene glycol presented this effect. Systems with preservatives showed stability in 90 days and had a slight influence on the particle size. The incorporation of nanosystems into three prototypes of matrices was proved by infrared spectroscopy and microscopy imaging. Antiradical properties were evaluated by radical scavenging-EPR spectroscopy. The strongest synergy NP, NE, and matrix were observed in the emulsion. The effect was observed and confirmed by an application study. Cream tested on volunteers increased skin hydration, elasticity, and had a smoothening effect. In regard to wrinkles, it reduced their area and depth. 

## Figures and Tables

**Table 1:** Nanoemulsion and nanoparticles of levan-stability test.

Sample	D0						D30						D90					
	Nanoemulsion System																	
	T [°C]						T [°C]						T [°C]					
	4		22		37		4		22		37		4		22		37	
	pH	O	pH	O	pH	O	pH	O	pH	O	pH	O	pH	O	pH	O	pH	O
control	6.04	A	6.28	A	6.38	A	6.3	A	4.48	A	6.9	D	6.50	A	4.60	A	7.05	E
A *	3.77	A	3.74	A	3,52	A	3.54	A	3.43	A	3.4	A	3.54	A	3.43	A	3.4	A
B	6.01	C	6.06	C	6.04	C	F						F					
C	6.11	C	6.22	C	6.21	C	F						F					
D	5.98	C	5.94	C	6.19	C	F						F					
E	5.93	C	5.93	C	6.06	C	F						F					
F *	6.04	A	6.01	E	6.01	E	5.64	E	5.94	C	5.90	A	5.64	E	5.94	B	5.9	A
G *	7.0	A	7.05	A	7.14	A	4.67	A	4.46	A	4.68	A	4.78	A	4.56	A	4.77	A
H	6.13	C	6.08	C	6.11	C	F						F					
I	5.19	B	5.24	B	5.08	B	F						F					
J	5.46	B	5.26	B	5.54	B	F						F					
K *	7.06	A	7.17	A	7.1	A	6.95	A	6.86	A	4.78	A	7.10	A	6.99	A	4.80	A
L *	7.39	A	7.32	A	7.39	A	6.99	A	7.28	A	6.99	A	7.08	A	7.40	A	7.08	A
M	3.8	C	3.39	C	3.6	C	F						F					
N *	4.26	A	4.31	A	4.27	A	4.5	A	4.23	A	4.25	A	4.65	A	4.29	A	4.36	A
O	6.19	C	6.17	C	6.28	C	F						F					
P	4.49	C	4.48	C	4.4	C	F						F					
R *	5.98	A	6.18	A	6.22	A	4.65	A	5.0	A	4.73	A	4.72	A	5.12	A	4.83	A
S	5.98	B	5.8	B	5.93	B	F						F					
T	5.57	B	5.55	B	5.55	B	F						F					
W *	5.95	A	4.87	A	5.99	A	5.97	A	4.82	A	5.96	A	6.09	A	4.92	A	6.08	A
Z *	5.77	A	5.79	A	5.83	A	5.79	A	5.82	A	5.86	A	5.91	A	5.94	A	5.98	A
AA *	5.94	A	5.43	A	6.09	A	5.96	A	5.48	A	6.04	A	6.08	A	5.59	A	6.16	A
AB *	5.92	A	4.93	A	5.97	A	5.96	A	4.97	A	5.91	A	6.08	A	5.07	A	6.03	A
AC	5.97	B	5.96	B	5.94	B	F						F					
AD *	5.95	A	5.17	A	6.23	A	5.97	A	5.23	A	6.27	A	6.09	A	5.33	A	6.40	A
AE *	5.98	A	5.35	A	6.1	A	5.99	A	5.37	A	6.12	A	6.12	A	6.11	A	5.48	A
**Nanoparticles of Levan System**																		
control	-	-	7.18	C	-	-	-	-	4.51	C	-	-	-	-	4.27	C	-	-
A	-	-	6.44	C	-	-	-	-	6.27	C	-	-	-	-	5.20	C	-	-
F	-	-	7.03	A	-	-	-	-	6.30	A	-	-	-	-	4.94	E	-	-
G	-	-	7.05	A	-	-	-	-	6.43	E	-	-	-	-	6.40	C	-	-
R	-	-	7.00	A	-	-	-	-	4.58	E	-	-	-	-	4.27	E	-	-
W	-	-	7.04	A	-	-	-	-	5.47	E	-	-	-	-	4.73	E	-	-
Z	-	-	6.96	A	-	-	-	-	6.99	A	-	-	-	-	6.83	A	-	-
AA	-	-	7.03	A	-	-	-	-	7.06	A	-	-	-	-	7.00	A	-	-
AB	-	-	7.00	A	-	-	-	-	7.01	A	-	-	-	-	6.11	A	-	-
AD	-	-	7.04	A	-	-	-	-	7.07	A	-	-	-	-	6.40	A	-	-
AE	-	-	7.04	A	-	-	-	-	5.91	C	-	-	-	-	5.08	E	-	-

(O) observation, (A) no changes (pass test), (B) phase separation, (C) sedimentation, (D) precipitation, (E) milky, (F) no further observations; * antimicrobials used in preservation efficacy test (Challenge test).

**Table 2:** DLS analysis of nanocarriers with the preservatives.

	Day 0			Day 90		
	D_H_ [nm]	PdI	ξ [mV]	D_H_ [nm]	PdI	ξ [mV]
Sample	Nanoemulsion System					
Control	110.5 ± 1.9	0.220 ± 0.01	−27.5 ± 2.83	120.2 ± 1.2	0.212 ± 0.010	−12.80 ± 1.18
A	165.3 ± 0.5	0.190 ± 0.003	40.34 ± 0.84	169.0 ± 3.0	0.195 ± 0.004	38.27 ± 1.26
F	385.2 ± 26.6	0.430 ± 0.01	−18.04 ± 0.42	195.7 ± 7.5	0.650 ± 0.120	−12.84 ± 0.40
G	146.6 ± 5.0	0.220 ± 0.01	−47.27 ±3.67	162.4 ± 1.1	0.094 ± 0.010	−22.20 ± 3.34
K	155.3 ± 0.9	0.190 ± 0.001	−31.60 ± 0.37	55.4 ± 10.9	0.591 ± 0.050	−14.07 ± 1.36
L	143.9 ± 7.4	0.210 ± 0.01	−39.00 ± 4.29	91.6 ± 5.5	0.636 ± 0.040	−26.10 ± 1.93
N	163.5 ± 3.3	0.220 ± 0.01	−4.92 ± 0.37	212.1 ± 1.7	0.280 ± 0.020	−7.41 ± 0.10
R	194.1 ± 0.8	0.240 ± 0.001	−20.07 ± 0.17	165.2 ± 1.4	0.130 ± 0.010	−16.13 ± 0.46
W	173.9 ± 1.1	0.160 ± 0.02	−29.34 ± 1.67	153.8 ± 1.2	0.190 ± 0.003	−15.97 ± 0.42
Z	212.8 ± 5.3	0.190 ± 0.02	−25.10 + 0.71	741.1 ± 19.5	0.199 ± 0.020	−9.88 ± 0.28
AA	184.2 ± 3.1	0.18 ± 0.20	−24.53 ± 1.72	256. 7 ± 3.6	0.116 ± 0.003	−12.10 ± 0.35
AB	165.9 ± 7.9	0.171 ± 0.02	−31.07 ± 1.89	160.8 ± 2.9	0.193 ± 0.005	−16.53 ± 0.40
AD	175.1 ± 1.0	0.180 ± 0.01	−28.93 ± 0.19	177.2 ± 2.6	0.181 ± 0.020	−15.23 ± 0.50
AE	487.3 ± 12.8	0.230 ± 0.01	−6.46 ± 0.15	169.0 ± 0.8	0.243 ± 0.004	−16.80 ± 0.20
**Nanoparticles of Levan System**						
control	203.1 ± 5.3	0.280 ± 0.001	−11.37 ± 2.17	311.1 ± 3.6	0.441 ± 0.01	−8.53 ± 0.95
AA	236.6 ± 3.9	0.115 ± 0.01	−4.68 ± 0.19	224.3 ± 4.6	0.136 ± 0.02	−1.07 ± 0.35

**Table 3:** List of used preservatives.

Preservative Symbol	INCI	pH Range	Max. Content [%]
	Pentylene Glycol, Glycerin, *Citrus Aurantium Amara* Fruit Extract, *Citrus Reticulata* Fruit Extract, *Citrus Aurantium Dulcis* Peel Extract, Ascorbic Acid, Citric Acid, Lactic Acid	3–6.5	3
**AA**	Glycerin + Propylene Glycol	unlimited	10 + 5
**AB**	Pentylene Glycol	2–12	5
**AC**	Phenethyl Alcohol	unlimited	1
**AD**	Butylene Glycol	unlimited	10
**AE**	Butylene Glycol + 1.2 Hexanediol	unlimited	1.5
**B**	Phenoxyethanol, Ethylhexylglicerin	<12	1
**C**	Benzyl Alcohol, Benzoic Acid, Dehydroacetic Acid	<6	1.2
**D**	Phenoxyethanol, Methylparaben, Ethylparaben, Propylparaben, Butylparaben	<8	1.2
**E**	Phenoxyethanol, Methylparaben, Ethylparaben, Propylene Glycol	to 8	1.4
**F**	Pentylene Glycol, Phenylpropanol	3–10	3
**G**	Potassium Sorbate, Sodium Benzoate	to 5.5	1.5
**H**	Phenylpropanol	3–10	1
**I**	Pentylene Glycol, Caprylyl Glycol, Ethylhexylglycerin	3–9	2
**J**	Benzyl Alcohol, Glyceryl Caprylate, Glyceryl Undecylenate	4–8	2
**K**	Sodium Levulinate, Sodium Benzoate	<6	2.5
**L**	Sodium Levulinate, Potassium Sorbate	<6	2
**M**	Benzyl Alcohol, Glyceryl Caprylate, Benzoic Acid, Propylene Glycol	4–6	1.2
**N**	Levulinic Acid, Glycerin, Sodium Levulinate	6	1
**O**	Pentylene Glycol, Glyceryl Caprylate, Glyceryl Undecylenate	3.5–7.5	2.5
**P**	Benzyl Alcohol, Dehydroacetic Acid	3–7	1
**R**	1.2-Hexanediol	unlimited	2.5
**S**	Caprylyl Glycol	unlimited	0.5
**W**	Propylene Glycol	unlimited	8
**Z**	Glycerin	unlimited	20

**Table 4:** Ingredients of the emulsion (face cream- C1) formulation.

Phase	INCI	Function	Content [%]			
			C	C1	C2	C3
**A**	Polyglyceryl-3 Dicitrate/Stearate	Emulsifier	2	2	2	2
	Caprylic/ Capric Triglyceride	Emollient	3.2	3.2	3.2	3.2
	Octyldodecanol	Emollient	2	2	2	2
	Triheptanoin	Emollient	2	2	2	2
	*Butyrospermum Parkii* (Shea) Butter	Emollient	3.6	3.6	3.6	3.6
	Cetearyl Alcohol	Viscosity controlling, Emollient	2.5	2.5	2.5	2.5
	Glyceryl Stearate	Viscosity controlling, Emollient	1.8	1.8	1.8	1.8
	Oleic/Linoleic/Linolenic Polyglyceride	Emollient, co-emulsifier	1	1	1	1
	Squalane	Emollient	2	2	2	2
	Tocopherol, *Helianthus Annuus* (Sunflower) Seed Oil	Antioxidant	0.1	0.1	0.1	0.1
	*Sesamum Indicum* Seed Oil	Emollient	1.5	1.5	1.5	1.5
**B**	Aqua	Solvent	40	40	40	40
	Sodium Phytate	Chelating agent	0.1	0.1	0.1	0.1
**C**	Microcrystalline Cellulose, Xanthan Gum	Viscosity controlling agent	0.9	0.9	0.9	0.9
	Glycerin	Humectant	0.5	0.5	0.5	0.5
	Aqua	Solvent	30	30	30	30
**D**	Propylene Glycol	Solvent	1.5	1.5	1.5	1.5
	Perfume	Parfum	0.1	0.1	0.1	0.1
	Water, Glycerin, *Punica Granatum* Fruit Extract, Potassium Sorbate, Sorbic Acid	Active ingredient	0.8	0.8	0.8	0.8
	Benzyl Alcohol, Benzoic Acid, Dehydroacetic Acid, Tocopherol	Preservative	0.8	0.8	0.8	0.8
**E**	Levan, Glucose, Fructose, Sucrose	Active ingredient	-	0.08	-	0.08
	Sodium Surfactin	Surfactant	-	0.375	0.375	-
	Diethylene Glycol Monoethyl Ether	Solvent	-	0.225	0.225	-
	Ascorbyl Tetraisopalmitate	Active ingredient	-	0.15	0.15	-
	Aqua	Solvent	to 100	to 100	to 100	to 100

**Table 5:** Ingredients of the face serum (S) formulation.

INCI	Function	Content [%]			
		S	S1	S2	S3
Propylene Glycol	Solvent	4.5	4.5	4.5	4.5
Pentylene Glycol	Solvent, Skin conditioning	3.5	3.5	3.5	3.5
Caprylyl/Capryl Glucoside	Surfactant	0.3	0.3	0.3	0.3
Glycerin	Humectant	0.8	0.8	0.8	0.8
Xanthan Gum	Viscosity controlling	0.95	0.95	0.95	0.95
Benzyl Alcohol, Benzoic Acid, Dehydroacetic Acid, Tocopherol,	Preservative	0.95	0.95	0.95	0.95
Perfume	Parfum	0.08	0.08	0.08	0.08
Levan, Glucose, Fructose, Sucrose	Active ingredient	-	0.08	-	0.08
Sodium Surfactin	Surfactant	-	0.375	0.375	-
Diethylene Glycol Monoethyl Ether	Solvent	-	0.225	0.225	-
Ascorbyl Tetraisopalmitate	Active ingredient	-	0.15	0.15	-
Aqua	Solvent	to 100	to 100	to 100	to 100

**Table 6:** Ingredients of the face tonic (T) formulation.

INCI	Function	Content [%]			
		T	T1	T2	T3
Betaine	Active Ingredient	1	1	1	1
*Aloe Barbadensis* Leaf Juice	Active ingredient	0.05	0.05	0.05	0.05
Sodium Phytate	Chelating agent	0.1	0.1	0.1	0.1
Perfume	Parfum	0.05	0.05	0.05	0.05
Propylene Glycol	Solvent	2	2	2	2
Sodium PCA	Active ingredient	1.5	1.5	1.5	1.5
Potassium Sorbate; Sodium Benzoate	Preservative	1	1	1	1
Levan, Glucose, Fructose, Sucrose	Active ingredient	-	0.08	-	0.08
Sodium Surfactin	Surfactant	-	0.375	0.375	-
Diethylene Glycol Monoethyl Ether	Solvent	-	0.225	0.225	-
Ascorbyl Tetraisopalmitate	Active ingredient	-	0.15	0.15	-
Aqua	Solvent	to 100	to 100	to 100	to 100

## Data Availability

The data presented in this study are available in supplementary material.

## Supplementary Materials

Figure S1. Images of vascular lesions and discoloration for the tested skin. Table S1. The initial number of microbial cells in the inoculum (N) and the initial number of cells found in 1g of the product tested (N0). Table S2. Demonstration of neutralizer efficacy. Table S3. Criteria for the evaluation of the preservation of cosmetic products. Table S4. The number of viable cells of test microorganisms after a given contact time with nanoemulsion and levan nanocarriers. Table S5. Log reduction values (Rx = lgN0−lgNx). Table S6. Skin moisturization effect. Table S7. Influence of skin firmness/elasticity. Table S8. Skin smoothening effect. Table S9. Reduction of wrinkles. Base cream. Table S10. Reduction of wrinkles. Formulations with nanosystems. Table S11. Microbial limits for cosmetic products according to EN ISO 17516: 2014. Table S12. Media used to assess the effectiveness of product preservation. SM 1. Microbiological analysis, SM 2. Evaluation of microbial protection: criteria and experimental conditions. SM. 3 Method used for inoculum enumeration. SM. 4 Preparation of bacterial suspensions and their incorporation into the tested formulations. SM. 5. Determination of the effectiveness of a preservative.
