# Supplementary material for: Targeted Hybrid Nanocarriers as a System Enhancing the Skin Structure
Source: Molecules. 2021 Feb 18;26(4):1063. doi: 10.3390/molecules26041063 (PMC7923190; doi:10.3390/molecules26041063)
Supplement: Supplementary file 1 [file molecules-26-01063-s001.pdf]

Article

Targeted Hybrid Nanocarriers as a System Enhancing the Skin Structure

Agnieszka Lewińska [1,*], Marta Domżał-Kędzia [2], Kinga Kierul [3], Michał Bochynek [2], Dominika Pannert [3], Piotr Nowaczyk [4,5] and Marcin Łukaszewicz [2,*]

[1] Faculty of Chemistry, University of Wroclaw, Joliot-Curie 14, 50-383 Wroclaw, Poland
[2] Department of Biotransformation, Faculty of Biotechnology, University of Wroclaw, Joliot-Curie 14, 50-383 Wroclaw, Poland; marta.domzal@uwr.edu.pl (M.D.-K.); michal.bochynek@uwr.edu.pl (M.B.)
[3] InventionBio Sp. z o.o., Wojska Polskiego 65 st., 85-825 Bydgoszcz, Poland, kinga.kierul@inventionbio.pl (K.K.); dominika.pannert@inventionbio.pl (D.P.)
[4] Faculty of Health Science, University of Opole, ul. Katowicka 68, 45-060 Opole, Poland; piotr.nowaczyk@uni.opole.pl
[5] Dr. Nowaczyk Research and Innovation Center Sp. z o.o. Sp. K., ul. Żmigrodzka 81-83 lok. 205, 51-130 Wrocław, Poland
* Correspondence: agnieszka.lewinska@uwr.edu.pl (A.L.); marcin.lukaszewicz@uwr.edu.pl (M.Ł.)

Table SM1. The initial number of microbial cells in the inoculum (N) and the initial number of cells found in 1g of the product tested (N_0).

Test strain inoculum	The initial number of microorganisms in suspension cfu/mL (N)	The initial number of cells in 1g of the test product cfu/mL (N_0)
E. coli (ATCC 8739)	1.02×10^8	1.02×10^6
S. aureus (ATCC 6538)	9.3×10^7	9.3×10^5
C. albicans (ATCC 10231)	9.6×10^6	9.6×10^4

Table SM2. Demonstration of neutralizer efficacy.

Strain	Number of CFU (CFU/ml) N_v (dilution 10^0)	A mixture of the neutralizer and diluent N_{vn} (dilution 10^0)	A mixture of the neutralizer and sample N_{vf} (dilution 10^0)	0,5 x N_{vn}
E. coli (ATCC 8739)	135	120	135	68
S. aureus (ATCC 6538)	187	201	206	103
C. albicans (ATCC 10231)	89	92	83	42

For all tested strains, the effectiveness of the neutralizer was above 50%, i.e. the condition $N_{vf} \geq 0.5\ N_{vn}$ was met, and the number of microorganisms from the control sample (N_{vn}) was - as required - similar to the amount of the N_v strain.

Table SM3. Criteria for the evaluation of the preservation of cosmetic products.

Microorganisms	Bacteria			Yeast		
**Sample Timing (days)**	**7**	**14**	**28**	**7**	**14**	**28**
**Criteria A**	A	≥3 and NI[2]	≥3 and NI	A	≥1 and NI2	≥1 and NI2
**Criteria B**	A	≥3	≥3 and NI	A	≥1	≥1 and NI2

A Not performed, 1 in this test, an acceptable range of deviation of 0,5 log is accepted, 2 NI – no increase in the count from the previous contact time, 3 $R_x = 0$ when $lgN0 = lgN_x$ (no increase from the initial count).

Table SM4. The number of viable cells of test microorganisms after a given contact time with nanoemulsion and levan nanocarriers.

		Preservative Symbol												
	**N_0 (CFU/ml)**	**A**	**AA**	**AB**	**AD**	**AE**	**F**	**G**	**K**	**L**	**N**	**R**	**W**	**Z**
		**NANOEMULSION**												
**Microbial strains**		**N14 (contact time)**												
E.coli	1.02×10^6	0	NC	0	NC	0	0	740	1030	NC	0	0	NC	1480

S. aureus	9.3×10^5	0	NC	0	NC	0	0	0	0	NC	0	0	NC	2930
C. albicans	9.6×10^4	0	NC	0	NC	0	0	0	190	NC	0	0	NC	980
		**N28 (contact time)**												
E.coli	1.02×10^6	0	NC	0	NC	0	0	380	850	NC	0	0	NC	2090
S. aureus	9.3×10^5	0	NC	0	NC	0	0	0	0	NC	0	0	NC	4000
C. albicans	9.6×10^4	0	NC	0	NC	0	0	0	0	NC	0	0	NC	280
**LEVAN NANOCARRIER**														
**Microbial strains**		**N14 (contact time)**												
E.coli	1.02×10^6	-	0	-	-	-	-	-	-	-	-	-	-	-
S. aureus	9.3×10^5	-	0	-	-	-	-	-	-	-	-	-	-	-
C. albicans	9.6×10^4	-	0	-	-	-	-	-	-	-	-	-	-	-
		**N28 (contact time)**												
E.coli	1.02×10^6	-	0	-	-	-	-	-	-	-	-	-	-	-
S. aureus	9.3×10^5	-	0	-	-	-	-	-	-	-	-	-	-	-
C. albicans	9.6×10^4	-	0	-	-	-	-	-	-	-	-	-	-	-

NC not countable.

Table SM5. Log reduction values ($R_x = lgN_0 - lgN_x$).

	**Preservative Symbol**												
	**A**	**AA**	**AB**	**AD**	**AE**	**F**	**G**	**K**	**L**	N	R	W	Z
**NANOEMULSION**													
**Microbial strains**	**Rx N14**												
E.coli	6	2	6	2	6	6	3	3	2	6	6	2	3
S. aureus	6	2	6	2	6	6	6	6	2	6	6	2	3
C. albicans	5	1	5	1	5	5	5	3	1	5	5	1	2
	**Rx N28**												
E.coli	6	2	6	2	6	6	3	3	2	6	6	2	3
S. aureus	6	2	6	2	6	6	6	6	2	6	6	2	2
C. albicans	5	1	5	1	5	5	5	5	1	5	5	1	3
**LEVAN NANOCARRIER**													
**Microbial strains**	**Rx N14**												
E.coli	-	6	-	-	-	-	-	-	-	-	-	-	-
S. aureus	-	6	-	-	-	-	-	-	-	-	-	-	-
C. albicans	-	5	-	-	-	-	-	-	-	-	-	-	-
	**Rx N28**												
E.coli	-	6	-	-	-	-	-	-	-	-	-	-	-
S. aureus	-	6	-	-	-	-	-	-	-	-	-	-	-
C. albicans	-	5	-	-	-	-	-	-	-	-	-	-	-

Table SM6. Skin moisturization effect.

Volunteer	Sex	First Measurement before Application (T0)	Second Measurement after Application (T1) Formulation C	Second Measurement after Application (T2) Formulation C1	Difference (T1 – T0)	Difference (T2 – T0)
1.	F	35.5 ± 0.3	37.6 ± 0.2	39.9	2.1	4.4
2.	F	35.9 ± 0.4	38.2 ± 0.4	40.8 ± 0.6	2.3	4.9
3.	F	36.7 ± 0.3	37.9 ± 0.4	42.3 ± 0.6	1.2	5.6
4.	F	37.3 ± 1.2	40.0 ± 1.3	42.5 ± 0.8	2.7	5.2
5.	F	36.0 ± 0.2	39.2	42.5 ± 0.2	3.2	6.5
6.	F	36.7 ± 0.5	38.7 ± 0.3	40.8 ± 1.1	2.0	4.1
7.	F	37.4	39.6 ± 0.7	42.4	2.2	5.0
8.	F	36.3 ± 1.1	38.4	42.5 ± 1.0	2.1	6.2
9.	F	35.8 ± 0.7	38.8 ± 0.2	43.0	3.0	7.2
10.	F	34.3 ± 1.6	38.0 ± 0.3	38.8 ± 2.1	3.7	4.5
Mean value		**36.2**	**38.6**	**41.6**	**2.4**	**5.4**

Assumption: an increase in the measurement value over time means greater skin moisturization.

Table SM7. Influence of skin firmness/elasticity.

Volunteer	Sex	First Measurement before Application(T0)	Second Measurement after Application(T1) Formulation C	Second Measurement after Application(T2) Formulation C1	Difference (T1 – T0)	Difference (T2 – T0)
1.	F	36.0 ± 0.2	36.6 ± 0.5	36.9 ± 0.6	0.6	0.9
2.	F	35.4 ± 0.4	37.8 ± 0.7	37.8 ± 0.1	2.4	2.4
3.	F	35.6	37.0 ± 0.5	37.7 ± 0.1	1.4	2.1
4.	F	36.2 ± 0.9	37.0	36.9 ± 0.5	0.8	0.7
5.	F	35.4	36.9 ± 2.0	36.9	1.5	1.5
6.	F	35.9 ± 0.1	38.0 ± 0.2	38.7 ± 0.8	2.1	2.8
7.	F	35.5 ± 1.1	37.8	38.3 ± 1.3	2.3	2.8
8.	F	36.7 ± 0.1	38.1 ± 0.8	38.7 ± 0.1	1.4	2.0
9.	F	36.0 ± 1.3	39.9 ± 0.3	39.7 ± 2.9	3.9	3.7
10.	F	37.3 ± 0.5	39.0 ± 0.7	40.0 ± 0.9	1.7	2.7
Mean value		36.0	37.8	38.2	1.8	2.2

Assumption: an increase in the measurement value over time means greater skin firmness/elasticity.

Table SM8. Skin smoothening effect.

Volunteer	Sex	Skin Firmness (px) before Application (T0)	Skin Firmness (px) after Application(T1) Formulation C	Second Measurement after Application(T2) Formulation C1	Skin Firmness (px) Difference (T1 – T0)	Skin Firmness (px) Difference (T2 – T0)
1.	F	11.0	10.0 ± 1.0	10.0	−1.0	−1.0
2.	F	12.0 ± 1.2	11.0	10.0 ± 1.2	−1.0	−2.0
3.	F	12.0 ± 1.0	12.0 ± 1.5	12.0 ± 1.0	0	0
4.	F	12.0	11.0 ± 0.6	11.0	−1.0	−1.0
5.	F	13.0 ± 2.3	12.0	11.0 ± 1.2	−1.0	−2.0
6.	F	11.0	10.0 ± 1.5	10.0 ± 1.2	−1.0	−1.0
7.	F	12.0 ± 2.0	12.0	12.0	0	0
8.	F	12.0 ± 1.2	12.0 ± 1.0	12.0 ± 1.0	0	0
9.	F	13.0	12.0	12.0	−1.0	−1.0
10.	F	11.0 ± 1.7	10.0 ± 1.7	10.0 ± 2.1	−1.0	−1.0
Mean value		11.9	11.2	11.0	−0.7	−0.9

Assumption: a decrease in the measurement value over time means an increase in the smoothness of the epidermis.

Table SM9. Reduction of wrinkles. Base cream.

Volunteer	Sex	The Volume of Wrinkles (px3) Before Application (T0)	The Volume of Wrinkles (px3) After Application (T1)	The Volume of Wrinkles Difference (T1 – T0)	Wrinkles Surface (px2) Before Application (T0)	Wrinkles Surface (px2) After Application (T1)	Wrinkles Surface Difference (T1 – T0)	The Depth of Wrinkles (px) (Before Application) (T0)	The Depth of Wrinkles (px) (After Application) (T1)	The Depth of Wrinkles Difference (T1 – T0)
1.	F	97452	96001	−1451	7849	7701	−148	12.0	11.0	−1.0
2.	F	97225	95293	−1932	7855	7628	−227	12.0	12.0	0
3.	F	99567	97449	−2118	7931	7703	−228	11.0	10.0	−1.0
4.	F	98473	96332	−2141	7820	7519	−301	12.0	11.0	−1.0
5.	F	96201	95141	−1060	7744	7611	−133	12.0	12.0	0
6.	F	97662	95214	−2448	7900	7716	−184	13.0	12.0	−1.0
7.	F	96778	95002	−1776	7846	7594	−252	12.0	12.0	0
8.	F	100258	97391	−2867	7922	7659	−263	11.0	11.0	0
9.	F	99364	96888	−2476	7833	7600	−233	11.0	10.0	−1.0
10.	F	97555	95719	−1836	7749	7518	−231	12.0	11.0	−1.0
Mean value		**98054**	**96043**	**−2011**	**7845**	**7625**	**−220**	**11.8**	**11.2**	**−0.6**

Table SM10. Reduction of wrinkles. Formulations with nanosystems.

Volunteer	Sex	The Volume of Wrinkles (px3) Before Application(T0)	The Volume of Wrinkles (px3) After Application (T1)	The Volume of Wrinkles Difference (T1 − T0)	Wrinkles Surface (px2) Before Application(T0)	Wrinkles Surface (px2) After Application (T1)	Wrinkles Surface Difference(T1 − T0)	The Depth of Wrinkles (px) Before Application) (T0)	The Depth of Wrinkles (px) After Application) (T1)	The Depth of Wrinkles Difference (T1 − T0)
1.	F	97420	95752	−1668	7856	7677	−179	12.0	12.0	0
2.	F	97239	95001	−2238	7870	7601	−269	12.0	11.0	−1.0
3.	F	99555	97001	−2554	7946	7640	−306	11.0	10.0	−1.0
4.	F	98443	95743	−2700	7805	7479	−326	11.0	10.0	−1.0
5.	F	96210	95012	−1198	7713	7580	−133	12.0	12.0	0
6.	F	97678	95123	−2555	7909	7697	−212	12.0	11.0	−1.0
7.	F	96760	94706	−2054	7855	7590	−265	12.0	11.0	−1.0
8.	F	100240	97111	−3129	7914	7663	−251	11.0	11.0	0
9.	F	99347	96890	−2457	7820	7640	−180	11.0	11.0	0
10.	F	97559	95640	−1919	7779	7407	−372	12.0	11.0	−1.0
Mean value		**98045**	**95798**	**−2247**	**7847**	**7597**	**−250**	**11.6**	**11.0**	**−0.6**

Table SM11. Microbial limits for cosmetic products according to EN ISO 17516: 2014.

Types of Microorganisms	Other Products
Total Aerobic Mesophilic Microorganisms (Bacteria plus yeast and mold)	≤ 1 × 10^3 CFU per g or ml
Escherichia coli	Absence in 1 g or 1 ml
Pseudomonas aeruginosa	Absence in 1 g or 1 ml
Staphylococcus aureus	Absence in 1 g or 1 ml
Candida albicans	Absence in 1 g or 1 ml

Table SM12. Media used to assess the effectiveness of product preservation.

Medium	Composition
**Tryptic Soy Agar (TSA)**	Pancreatic digest of casein 15 g/L, papaic digest of soybean meal 5 g/L, sodium chloride 5 g/L, agar 15 g/L; Final pH 7.3 +/- 0.2 at 25 °C
**D/E Neut Broth**	Dextrose 10 g/L, Lecithin 7 g/L, Sodium Thiosulfate 6 g/L, Pancreatic Digest of Casein 5 g/L, Tween® 80 5 g/L, Yeast Extract 2.5 g/L, Sodium Bisulfite 2.5 g/L<br>Sodium Thioglycollate 1 g/L, Monopotassium Phosphate 0.1 g/L, Bromcresol Purple 0.02 g/L; Final pH 7.6 ±0.3 at 25 °C
**Sabouraud Dextrose Agar (SDA)**	Glucose 40 g/L, a peptic digest of animal tissue 5 g/L, a pancreatic digest of casein 5 g/L, agar 15 g/L

SM 1. Microbiological analysis

Preserved nanosystems were subjected to microbiological tests. Namely, 1 g of preserved nanosystem was 10 times diluted with neutralizing broth DE (Dey/Engley broth, Oxoid) and incubated at room temperature for 30 min. Such prepared dilution was pipetted on a Petri dish and 15 - 20 ml of melted agar was poured, the temperature of agar not exceeding 48 °C. Petri dishes were gently rotated to disperse the sample. For detection of mesophilic bacteria, Tryptic Soy Agar (TSA, Oxoid) was used. For detection of yeast and molds Sabouraud Dextrose Agar (SDA, Oxoid) was used. Plates were incubated for 72 h at 25 ± 2.5 °C.

SM 2. Evaluation of microbial protection: criteria and experimental conditions

Preservation efficiency test (Challenge test) was performed according to the method described in normative document ISO 11930. Nanocarrier systems have been subjected to stress tests in order to assess the effectiveness of preservation (EN ISO 11930: 2012 Cosmetics - Microbiology - Evaluation of the antimicrobial protection of a cosmetic product (ISO 11930: 2012)). Obtained results were assessed based on criteria in Table SM3.

Challenge test was conducted using two bacterial strains *Escherichia coli* (ATCC 8739), *Pseudomonas aeruginosa* (ATCC 9027), and yeast strain *Candida albicans (ATCC 10231).* Bacterial suspensions were obtained in a way that reaches $1 \times 10^5 – 1 \times 10^6$ of Colony Forming Units (CFU) for bacteria and $1 \times 10^4 – 1 \times 10^5$ CFU for yeast in tested formulations. All microbial media used during the test are listed in Table SM 12.

Bacteria were incubated at 30 - 35 °C for 48 – 72 hours.

SM. 3 Method used for inoculum enumeration

For the enumeration of viable microorganisms in the inoculum, the pour plate method was used: 1 ml of the appropriate decimal dilution was applied to the Petri dish and subsequently 15 - 20 ml of melted agar medium was poured.

SM. 4 Preparation of bacterial suspensions and their incorporation into the tested formulations

To prepare the infecting inoculum of the test strain, the loopful of bacterial cells grown on an agar plate was transferred to a sterile tube containing 5 ml of the diluent D/E. Bacterial suspensions were set to contain $1 \times 10^7 – 1 \times 10^8$ CFU, for yeast $1 \times 10^6 – 1 \times 10^7$ CFU. The exact number of cells in the infecting suspensions (N) has been determined, by the preparation of subsequent decimal dilutions.

On this basis, the initial number of cells in 1 g of the tested product (N_0) was calculated. The results are presented in Table SM 1.

SM. 5. Determination of the effectiveness of a preservative

To validate whether the applied diluent can neutralize the preservative without inhibiting the growth of microorganisms, the effectiveness of the neutralizer was determined. For this purpose, the number of microorganisms determined in the microbial suspension was incubated in the presence and absence of the test product and the neutralizer. The applied neutralizer is considered efficient when the recovery of microorganisms in the presence of the tested product is at least 50% concerning the number of microorganisms not treated with the tested product. The results of determining the effectiveness of the neutralizer are presented in Table SM 2.

SM. 6 Determination of the preservation efficacy

The number of viable microbial cells was assessed for all tested preservation systems for both, nanoemulsion and levan nanoparticles. Tests were prepared for samples at 14 and 28 days of contact, using the method mentioned in SM1. The test results are presented in Tables SM. 4 and SM. 5.

The study determined the number of cells of the test microorganisms after the specified contact times of the preparation with the inoculated test microorganisms. The contact times were 14 days and 28 days for bacteria and *C. albicans*, and 14 and 28 days for *A. brasiliensis*, respectively. To determine the number of live cells in 1 g of the tested product, inoculation was performed using the flooding method, using the diluent specified in PN-EN ISO 11930: 2012 standard. Before sowing in Petri dishes, the product was left in the diluent for 30 ± 15 minutes at room temperature. The test results are presented in Tables SM. 4 and SM. 5.

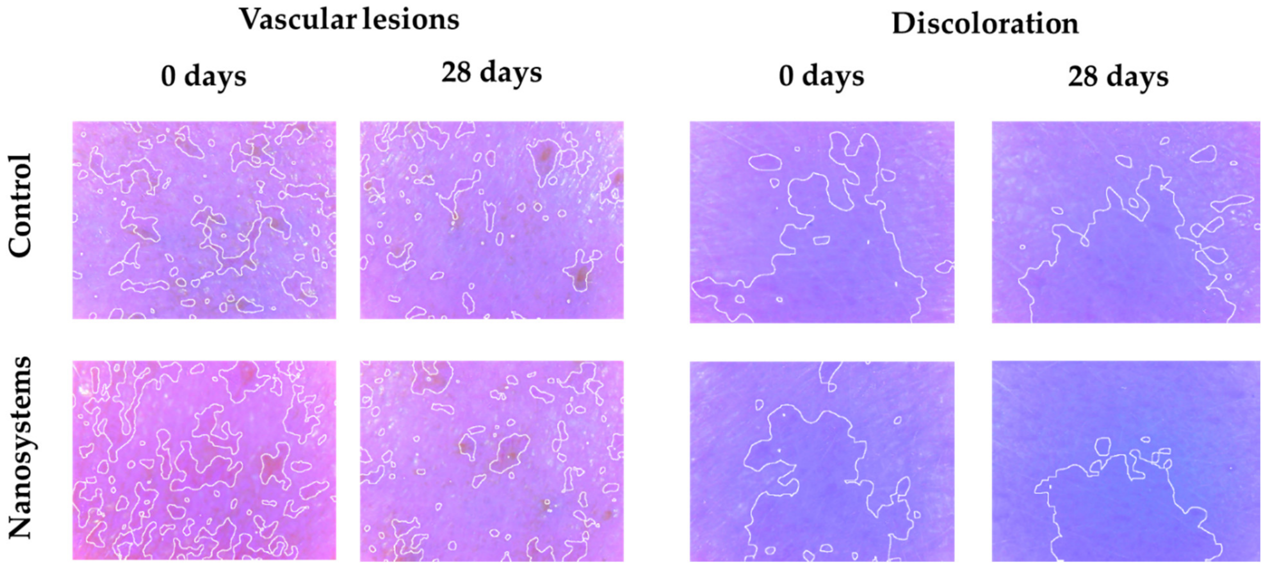

Figure SM1. Images of vascular lesions and discoloration for the tested skin.
